# Malnutrition in paediatric patients with leukaemia and lymphoma: a retrospective cohort study

**DOI:** 10.3332/ecancer.2021.1327

**Published:** 2021-12-02

**Authors:** Hardenson Rodríguez González, Sergio Andrade Mejía, Javier Orlando Contreras Ortiz, Adriana Patricia Osorno Gutiérrez, Jorge Eliécer Botero López, Javier Enrique Fox Quintana

**Affiliations:** 1 University of Antioquia, Carrera 51d #62-29, Medellín 050010, Colombia; 2 Paediatrics and Child Health Department, University of Antioquia, Calle Barranquilla #51b-22, Medellín 050010, Colombia; 3 An tioquia School of Engineering, Universidad EIA, Vda. El Penasco, Envigado, Antioquia 055428, Colombia; 4 San Vicente Children’s Hospital Foundation, Calle Barranquilla #51b-22, Medellín 050010, Colombia; a https://orcid.org/0000-0001-7027-7476; b https://orcid.org/0000-0001-5823-6110; c https://orcid.org/0000-0001-8568-5421; d https://orcid.org/0000-0003-3513-7659; e https://orcid.org/0000-0003-2907-5500; f https://orcid.org/0000-0002-1014-9402

**Keywords:** malnutrition, paediatric cancer, infections, mortality, nutritional assessment

## Abstract

**Introduction:**

Paediatric cancer is a potentially curable disease and its prognosis has been linked to several factors, such as nutritional status. The impact of malnutrition on these patients, either by overnutrition or undernutrition, varies and its relationship with outcomes is inconsistent. This study was conducted in order to determine the frequency of malnutrition in children with haematolymphoid malignancies at the time of diagnosis, as well as during treatment and to also investigate its relationship with the development of infections and death.

**Materials and Methods:**

A retrospective cohort study of 191 children with a recent diagnosis of a haematolymphoid malignancy. The risks and nutritional classification were determined using anthropometry, follow-ups were conducted for up to 24 months and the presentation and frequency of infections and/or death were also recorded. Bivariate and multivariate analyses were conducted using binomial logistic regressions, for death and infection outcomes during follow-up. Survival analysis was conducted for various factors and types of cancer.

**Results:**

83.7% of children had a sufficient nutritional classification at diagnosis, 6.8% had malnutrition by undernutrition and 9.4% by overnutrition. 83.8% had at least one infectious complication during follow-up and 47.1% had ≥ 3. This percentage increased to 69.2% when configuring it in the malnutrition by undernutrition group. 18.3% of patients died. When configuring the mortality, the percentage was greater in patients with Acute Myeloid Leukaemia (AML) (57.1%) and malnutrition by undernutrition (30.7%). The multivariate analysis for the outcome of death, only showed a statistically significant variable (AML odds ratio = 26.52; confidence interval = 1.09–643.24; *p* = 0.04).

**Conclusion:**

No statistically significant relationship was found between the nutritional status of children with haematolymphoid neoplasms, and outcomes such as infections or death. The differences in the results obtained in these investigations may be related to the varied nutritional status definitions and the ways of measuring them, thus limiting comparisons between them.

## Introduction

It is estimated that more than 400,000 new cases of paediatric cancer are diagnosed throughout the world each year [1], 80% of whom live in countries with limited resources [2]. Paediatric cancer is a potentially curable disease, with survival rates exceeding 80% in high-income countries, as opposed to low-income countries where it ranges between 15% and 45% [3]. It is responsible for significant morbidity and mortality in the paediatric population and is the second cause of death in children from countries with a high socio-demographic index (SDI) and an increasing number of middle SDI countries [4].

Progression of the disease itself is the main cause of the death in children with cancer, followed by treatment-related toxicity where infectious complications, especially chemotherapy-induced neutropenia, account for more than half of the cases [5]. There are several factors associated with unfavourable outcomes and death in these patients, including: the type of cancer, a lack of information about the disease, poor access to health services, delayed diagnosis and initial treatment as well as a lack of resources for comprehensive treatment and nutritional status [6].

Malnutrition in children with cancer, whether it be by overnutrition or undernutrition, has been linked to poor outcomes, greater rates of treatment discontinuation, hospital stays, treatment-related toxicity, infectious complications and mortality [7]. Hypotheses have been developed that suggest malnutrition impairs the ability of the immune response against infections due to cytokine hormone function alterations [8] and micronutrient deficiencies [9, 10]. It also impairs the effectiveness of cancer treatments because of pharmacokinetic and pharmacodynamic changes that are explained by an altered metabolic state [11]. Likewise, it has also been associated with a poorer quality of life and poorer physical, social and emotional performances [12]. Undernourishment is reported in 6% to 65% of cases in children with cancer and being overweight in 8% to 78%. The prevalence varies depending on the study population [13–16].

There are still questions concerning malnutrition in patients with paediatric cancer, including the causes, prevalence, impact on the disease and the relationship with complications and outcomes. Although there is increasing evidence in this respect, the studies provide varied information because of the heterogeneity in their design and study population [17–19].

As nutritional status is a factor that is subject to potential change, this study was conducted to determine the frequency of malnutrition in children with haematolymphoid malignancies at the time of diagnosis, as well as during treatment and to also investigate its relationship with the development of infectious complications and death. It thereby provides information that could lead to strategies that will impact the treatment of paediatric cancer.

## Methods

With the prior approval of the ethics committee at the San Vicente Children’s Hospital Foundation in Medellín, Colombia, a cohort study was conducted at said hospital, which reviewed the medical records of patients under 18 with haematolymphoid neoplasms. By opting to study all available cases in the database provided by the hospital under International Classification of Diseases 10: C77-C96.9, D46, D47, the patient selection was non-probabilistic for convenience. The information was provided using a prior pilot standardised format in Google Forms.

The sample size was estimated using the following parameters: 80% power (*β*), 0.05 precision (*α*) and a 20% malnutrition relationship. The sample size estimation was a total of 110 patients, 92 of whom had an adequate nutritional status and 18 had a malnutrition status. The calculations were made using the STATA v 10 statistical software.

Those that started and finished treatment between 2013 and 2020 were included and those that discontinued treatment, relapsed, received haematopoietic stem cell transplants, had comorbidities that affected the nutritional status like HIV, congenital or acquired immunodeficiencies, cardiopathy with haemodynamic compromise, swallowing disorders and those who died within the first 72 hours of beginning treatment were excluded.

Age, gender and type of insurance were recorded to characterise this population (in Colombia, the contributory regime applies to workers with the ability to pay and pensioners. The subsidised regime applies to the poor who have been registered and receive social assistance from the state, the affiliated population without the ability to pay who have not been registered, and the special regime for workers of the military forces, and national police amongst other such State entities) [20]. The specific diagnosis and risk classification were determined by the attending team. Lymphoblastic lymphoma was considered to be a dissimilar group to non-Hodgkin lymphoma in the records, owing to the fact the prognosis and chemotherapy protocols are similar to acute lymphoid leukaemia (ALL) [21]. The anthropometric assessment was obtained from the medical records, without knowing the calibration status of the teams or the training of the staff that obtained it. It was recorded at the time of the initial cancer diagnosis and in follow-up intervals of 6, 12, 18 and 24 months. The nutritional classification was conducted by only considering anthropometric indicators associated with weight and size and were obtained using the World Health Organization (WHO) Anthro tool [22] under the WHO. Undernourishment and serious undernourishment were then categorised as malnutrition by undernutrition, and being overweight and obesity as malnutrition by overnutrition. The nutritional risk was classified as per the type of diagnosis, with the model proposed by Rickard *et al* [23]. As for the outcomes, the time of death was recorded and the cause identified. The time the episodes of infection occurred was also recorded and were classified into categories of the most common and highest risk entities: febrile neutropenia (FN), serious infection (bacteraemia, catheter-associated infections, sepsis, pneumonia, pyelonephritis, meningitis, osteomyelitis, septic arthritis, serious soft tissue infections and neutropenic) amongst others [24].

The data were consolidated in an Excel database and analysed using the version 23 of SPSS software. Graphical representations and normality statistics were used for the descriptive statistics, using the Kolmogórov–Smirnov test. Variables with normal distribution were recorded using their standard deviation and mean and those that had asymmetrical distribution, with the median and percentiles 25 and 75. Qualitative nominal variables and categories were recorded as frequencies as well as their respective proportions. By considering age, type of cancer, chemotherapy schedules and nutritional classification by anthropometry as control variables, bivariate analyses were conducted to identify potential variables and multivariate analyses were subsequently conducted, with binomial logistic regression for the outcomes of death and infectious episodes during follow-up. Lastly, survival analyses were conducted for various factors and types of cancer.

## Results

**Figure 1.**
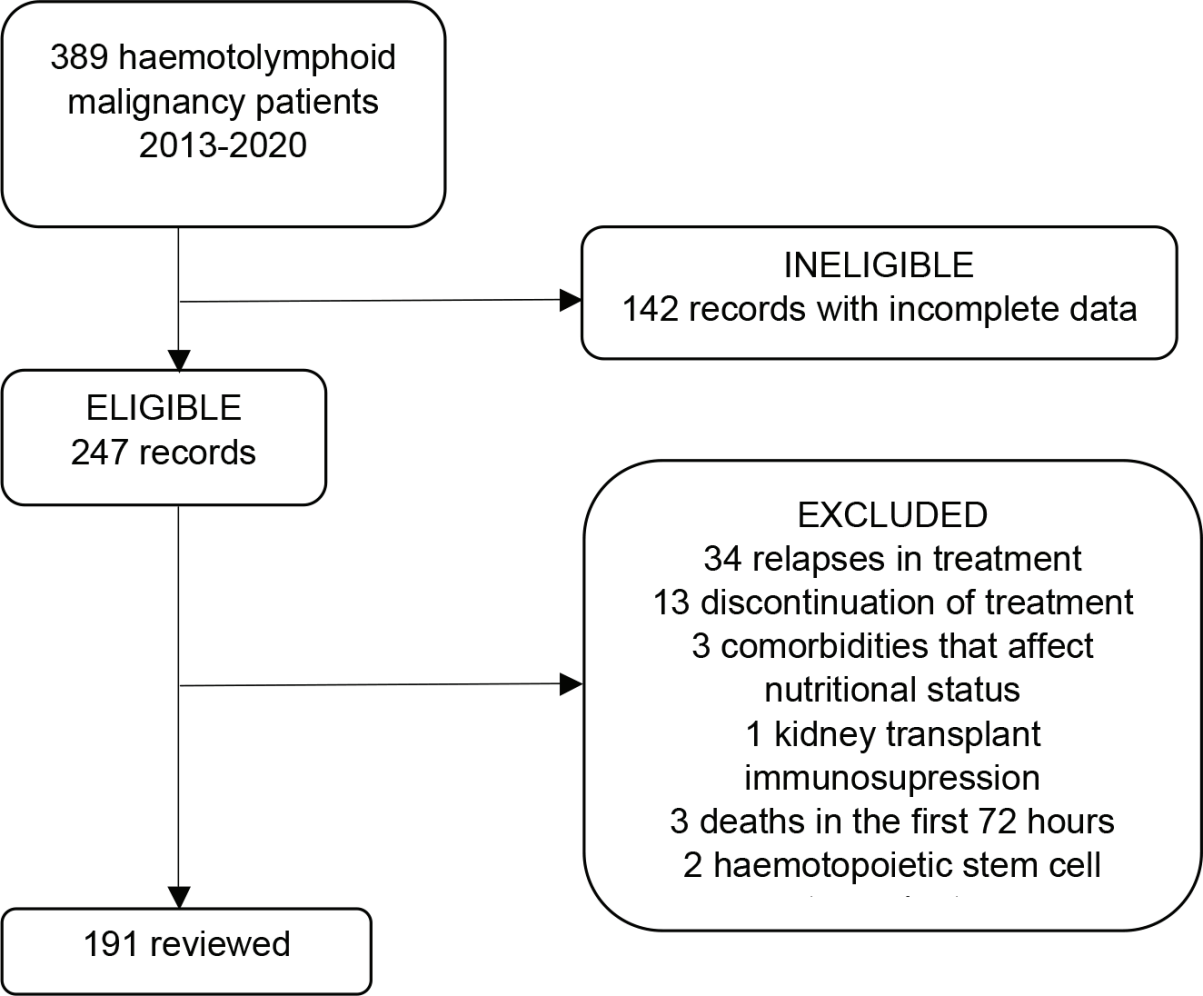
Medical records review.

Data for 191 patients were recorded upon selection of the records, as is explained in Figure 1.

The characteristics of the patients studied are shown in Table 1. We found a greater number of males, an average age of 6.28 years, a prevalence of subsidised insurance and a greater proportion of patients with ALL and high nutritional risk. The variable of interest, the nutritional status, was adequate for most patients with only 16.2% with a malnutrition status.

**Figure 2.**
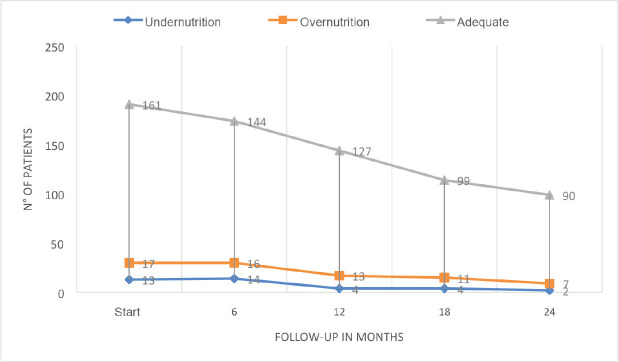
Nutritional status at diagnosis, and 6-month follow-up.

The developments in nutritional classification from initial diagnosis to months 6, 12, 18 and 24 of follow-up, are presented in Figure 2 and Table 2. The missing data is due to deaths and differences in follow-up times depending on diagnoses (6 to 12 months in lymphomas and up to 24 months in leukaemia).

The death occurred in 35 patients during follow-up (18.3%), most of which were due to infections (13%). When the mortality rates were configured to diagnosis type, age and nutritional status, greater frequencies were found in AML 57.1% and malnutrition by undernutrition 30.7% with regard to the sample total (Table 3).

553 infection episodes were recorded in 160 patients, 79.2% of whom (438) had FN. Serious infections were recorded in 49% of cases (216) during this episode. 20.7% (115) experienced a non-FN-related infection. Out of the entire patient group, 90 had 3 or more infection episodes (47%). This is presented in an age, diagnosis type and nutritional status-configured manner in Table 4, where there is an evident increase in the number of infections in children under 5, (66.6%), AML (64.2%), non-Hodgkin lymphoma (70%) and malnutrition by undernutrition (69.2%).

The multivariate analysis with logistical regression for the outcome of death, only showed a statistically significant association and this was in the AML type of cancer (odds ratio (OR) = 26.52; confidence interval (CI) = 1.09–643.24; *p* = 0.04) in a model that accounts for at least 50% of the mortality. No potential association was found for the outcome of three or more infection episodes. The interaction between variables, such as insurance company and cancer type, comorbidity and cancer type, initial nutritional status and cancer type, age and cancer type, was assessed for the outcome of death and no statistical association was found between these variables.

**Figure 3.**
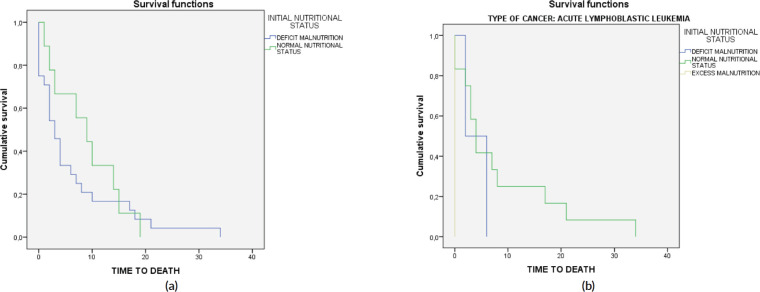
Survival analysis for the initial nutritional status. (a): In the entire sample. (b): In patients with ALL.

Lastly, a survival analysis was conducted that assessed the influence of various factors on mortality, including the initial nutritional status, type of cancer, type of insurance company and, in the case of the most common cancer (ALL), the relationship between the initial nutritional status and death (Figure 3). Statistically significant differences in mortality averages were only observed in the case of cancer type and death (Table 5).

## Discussion

It is widely recognised in the literature that the nutritional status of patients with cancer is affected by several intrinsic factors such as the type of cancer, location, the clinical stage or type of neoplastic therapy as well as extrinsic factors such as poverty, lack of education and poor access to health. The latter are of greater relevance in developing countries [25].

It was found in this current study that most patients had an adequate nutritional status at the time of diagnosis (83.7%), with a smaller proportion of patients with malnutrition by undernutrition (6.8%) and malnutrition by overnutrition (9.4%). These results concur with the information provided in high and middle income countries like the Orgel *et al* [26] cohort in the USA, which reported undernourishment in 5.8% and obesity in 13.9%, or that of Triarico *et al* [27] in Italy that reported mild undernourishment in 12.7%, moderate in 1.6% and serious in 3.1%. Similar to this investigation, malnutrition by overnutrition dominated over undernourishment in some of these studies, which may be related to the current problem of rising obesity [28].

When comparing these results with those obtained in studies from Central and South America, there are significant differences given that higher rates of undernourishment were reported at diagnosis. This includes the study conducted in Venezuela by Fuente *et al* [29] with malnutrition by undernutrition in 40.4% of children and by overnutrition in 10.5%, or that conducted in Colombia by Suarez *et al* [30] who reported acute undernourishment in 13.6%. The explanation for these differences may not only be attributed to socio-economic conditions. By using different strategies to assess the nutritional status, such as the triceps skinfold, the average arm circumference or biochemical markers like albumin; higher rates of undernourishment are recorded. This is evident in studies like that of Maia Lemos *et al* [31] in Brazil, where undernourishment was found at diagnosis in up to 27.3% of patients, that of Villanueva *et al* [32] in Guatemala that reported 47% or that of Peccatori *et al* [33] in Nicaragua that reported 65.4%. The latter coincides with the undernourishment percentages reported in low and low to middle-income countries, where it reaches up to 40%–90% [34, 35]. It is thereby important to emphasise that in the definition of malnutrition heterogeneity is a determinant of the variability reported in different studies, the methodology used to assess the nutritional status (anthropometric or biochemical measures) and the criteria or cut-off points that vary from population to population, thus making it difficult to accurately estimate the prevalence of cancer-related malnutrition [36]. This may also be the reason for undernourishment rates being low in this study, when compared with those reported in countries in Latin America.

The nutritional status of paediatric patients with cancer is changeable. It has been shown that the development of alterations may not only occur at the time of diagnosis, but also during treatment [37]. In the case of the study population, there was a slight increase in the number of patients with an adequate nutritional status during the 6-month follow-up at 24 months (84% to 90.9%), in relation to a reduced number of patients with malnutrition by undernutrition (6.8% to 2%) and malnutrition by overnutrition (8.9% to 7%). However, it should be made clear that these percentages do not reflect a consistent number of patients at all times, given that those affected by lymphomas were followed-up for 6 or 12 months and those affected by leukaemia for up to 24 months. Combining this with those lost from death, changes the understanding of this trend. The results outlined are similar to those reported in other countries, like those of Revuelta Iniesta *et al* [38], who followed patients for 36 months and found greater undernourishment at diagnosis than at any other time and an increase in malnutrition by overnutrition as time went by. This initial trend of further deterioration in the nutritional status, with subsequent recovery and an increase in the number of overweight patients, has also been reported in other investigations [39, 40]. This behaviour may be related to chemotherapies being less intensive as time goes by and treatment-related mortality (TRM) and hospitalisations also decreasing, thus enabling an improved nutritional status. This requires further investigation.

Several publications have identified the potential adverse effects of nutritional problems during treatment, including a reduced tolerance of chemotherapy, drug-altered metabolism, impaired immunity and increased TRM. The latter is shown in a greater number of complications, infections, relapses or death [41]. However, the quality of the evidence supporting each of these effects is varied [42] and conflicting in some cases [43, 44].

74.9% of patients was classified as high nutritional risk at diagnosis and 25.1% as low risk, without finding a statistical relationship with the outcomes. They used the STRONG kids scale in investigations like that of Yoruk *et al* [45] and found a moderate nutritional risk in 71.6% of patients at diagnosis and a high risk in 28.4%. The latter has an independent impact on infections (hazard ratio (HR) = 5.9, CI of 95% = 1.56–22.29 *p* = 0.009)), and differs from the findings of this investigation. Although the literature recommends assessing the nutritional risk in cancer patients to identify those who have a greater need for intervention, this is not a routinely reported practice in these studies. This may be explained by a lack of standardised and validated methods for the overall paediatric population [46], thus limiting the comparison of findings.

With regard to the relationship between the nutritional status and infections, it was found in this study that 83.8% of patients had at least 1 infectious complication and 47.1% had ≥ 3 during the follow-up period. This number increased in malnourished patients, albeit without a statistically significant relationship (OR = 0.951 CI 0.4–2.1 *p* = 0.906)). These results contrast with that reported by Loeffen *et al* [47], who found a strong association between rapid weight loss in the first 3 months of treatment and a higher rate of FN episodes with bacteraemia in the first year after diagnosis (OR = 3.05, CI of 95% = 1.27–7.30, *p* = 0.012). However, when it comes to the impact of these episodes, the investigations concur that there are no statistically significant differences between patients who were adequately nourished at the time of diagnosis, and patients with malnutrition by undernutrition or overnutrition. Similar results have been reported in investigations like that of Pribnow *et al* [48]. The current investigation did not assess weight loss over time in terms of percentage, but independently determined the nutritional status at all times. It combined heterogeneity in the study designs with various forms of measured outcomes (number of infectious events, severity or TRM), which limits the correlation between results.

An outcome of death occurred in 18.35% of patients, which increased to 30% when configuring in patients with malnutrition by undernutrition. A statistically significant relationship was only found with the AML type of cancer (OR = 26.52; CI = 1.09–643.24; *p* = 0.04). The relationship between undernourishment and clinical outcomes remains unclear [49]. Some investigations have found that there is an association with poorer results [50]. Triarico *et al* [27] reported that the risk of mortality increased by 294% in patients who lost ≥ 5% of weight over the 3-month period after diagnosis and by 110% in patients with a weight loss of ≥ 10% over 6 months, which contrasts with the findings of this and other investigations [51, 52]. As for obesity, no relationship was found with the outcome of death in this study, either. This association has been described inconsistently in several investigations with a particular interest in patients with ALL [53]. This had led to meta analyses being conducted, like that of Amankwah *et al* [54] who reported a greater risk of mortality with a higher body mass index (BMI) at the time of diagnosis (HR = 1.30, CI of 95% = 1.16–1.46). These results differ from those of Løhmann *et al* [55], who found no association between BMI at the time of diagnosis and the prognosis of children aged 2 to 9 years, but did find a trend towards better results in overweight children aged 10 to 17 years. The latter highlights the range of factors that may influence the nutritional status of patients with cancer, and leads to difficulties in obtaining conclusions that are applicable to the overall population.

Nutritional status has also been unpredictably associated with the risk of relapse [56]. With this being considered as a factor of further decline itself in the nutritional status, this outcome was not assessed in the study since these patients are excluded.

There are many limitations to understanding the results obtained in this study. Amongst them are the fact this was a retrospective investigation, which affects the accuracy of the information collected, a lack of technique standardisation, non-calibrated tools to obtain anthropometric measures and the nutritional status classification being limited to the use of certain WHO anthropometric indicators (weight and size). Others such as measuring the skinfold or biochemical markers are not included, since they are not part of the routine assessment, nor are clinical signs or food history given the retrospective nature of this study. This may be related to the low number of patients found to be malnourished, which directly affects its association with the outcomes. It is essential that the classification criteria for risk and nutritional status in patients with cancer are consolidated for the correlation between these results to be more reliable.

## Conclusion

No statistically significant relationship was found in this study between the nutritional status of children with haematolymphoid neoplasms and outcomes such as infections or death. The differences in results obtained in these investigations may be related to the varied nutritional status definitions and the ways of measuring it, which limits comparisons between them. Conducting a prospective and interdepartmental investigation would be ideal, in order to obtain a more representative sample with results that are applicable to the population.

## Conflicts of interest

No conflicts of interest were declared by the authors.

## Funding

No sources of funding were received.

## Figures and Tables

**Table 1.** Characteristics – children with haematolymphoid malignancy 2013–2020 (*n*: 191).

Age average 6.28, p25 3,85–p75 10.08	
Gender, *n* (%)	
Male	106 (55.5)
Female	85 (44.5)
Type of insurance, *n* (%)	
Subsidised	116 (60.7)
Contributory	63 (33)
Special	8 (4.2)
Affiliated	4 (2.1)
Type of cancer, *n* (%)	
ALL	110 (57.6)
Hodgkin lymphoma	39 (20.4)
Non-Hodgkin lymphoma	20 (10.5)
Acute myeloid leukaemia (AML)	14 (7.3)
Lymphoblastic lymphoma	8 (4.2)
Nutritional risk, *n* (%)	
High	143 (74.9)
Low	48 (25.1)
Nutritional status at onset *n* (%)	
Malnutrition by undernutrition	13 (6.8)
Adequate	160 (83.7)
Malnutrition by overnutrition	18 (9.4)

p25: percentile 25, p75: percentile 75

**Table 2.** Nutritional status at diagnosis, and 6-month follow-up.

		Follow-up time, *n* (%)				
		Diagnosis	6 months	12 months	18 months	24 months
Nutritional status	Under	13 (6.8)	14 (8.0)	4 (2.7)	4 (3.5)	2 (2)
	Adequate	161 (84.2)	144 (82.7)	127 (88.1)	99 (86.8)	90 (90.9)
	Over	17 (8.9)	16 (9.1)	13 (9)	11 (9.6)	7 (7)
Total, *n* (%)		191 (100)	174 (100)	144 (100)	114 (100)	99 (100)

**Table 3.** Mortality in children with haematolymphoid malignancy 2013–2020 (*n*: 191).

Death, *n* (%)	35 (18.3)
Infection	25 (13.1)
Infection and the progression of disease	4 (2.1)
The progression of disease	5 (2.6)
Central nervous system thrombosis	1 (0.5)
Clinical variable-configured mortality, *n* (%)	
Age, *n* (%)	
Under 5 (*n*: 66)	13 (19.6)
5 and over (*n*:125)	22 (17.6)
Type of diagnosis, *n* (%)	
AML (*n*: 14)	8 (57.1)
ALL (*n*: 110)	25 (22.7)
Lymphoblastic lymphoma (*n*: 8)	1 (12.5)
Non-Hodgkin lymphoma (*n*: 20)	1 (5)
Hodgkin lymphoma (*n*: 39)	0 (0)
Nutritional status, *n* (%)	
Malnutrition by undernutrition (*n*: 13)	4 (30.7)
Malnutrition by overnutrition (*n*: 18)	0 (0)
Adequate (*n*: 160)	31 (19.3)

**Table 4.** Distribution of patients with three or more infection episodes.

Age, *n* (%)	
Under 5 (*n*: 66)	44 (66.6)
Over 5 (*n*: 125)	46 (36.8)
Type of diagnosis, *n* (%)	
AML (*n*: 14)	9 (64.2)
ALL (*n*: 110)	63 (57.2)
Lymphoblastic lymphoma (*n*: 8)	2 (25)
Non-Hodgkin lymphoma (*n*: 20)	14 (70)
Hodgkin lymphoma (*n*: 39)	2 (5.12)
Nutritional status, *n* (%)	
Malnutrition by undernutrition (*n*: 13)	9 (69.2)
Adequate (*n*: 160)	74 (46.2)
Malnutrition by overnutrition (*n*: 18)	7 (38.8)

**Table 5.** Statistical associations in the analysis of survival.

Overall comparisons	Log rank (Mantel–Cox) Chi-squared test	*p*
Type of cancer (AML)	9.43	0.02
Initial nutritional status ALL	4.57	0.10
Initial nutritional status	0.49	0.48
Insurer	0.68	0.88
